# Prediction Model of Powdery Mildew Disease Index in Rubber Trees Based on Machine Learning

**DOI:** 10.3390/plants14152402

**Published:** 2025-08-03

**Authors:** Jiazheng Zhu, Xize Huang, Xiaoyu Liang, Meng Wang, Yu Zhang

**Affiliations:** 1 Sanya Institute of Breeding and Multiplication, Hainan University, Sanya 572025, China; zhujiazheng2022@163.com (J.Z.); h414423400@163.com (X.H.); liang2017@hainanu.edu.cn (X.L.); 2 School of Tropical Agriculture and Forestry, Hainan University, Danzhou 571737, China

**Keywords:** rubber tree, disease index, *Erysiphe quercicola*, predict model, machine learning

## Abstract

Powdery mildew, caused by *Erysiphe quercicola*, is one of the primary diseases responsible for the reduction in natural rubber production in China. This disease is a typical airborne pathogen, characterized by its ability to spread via air currents and rapidly escalate into an epidemic under favorable environmental conditions. Accurate prediction and determination of the prevention and control period represent both a critical challenge and key focus area in managing rubber-tree powdery mildew. This study investigates the effects of spore concentration, environmental factors, and infection time on the progression of powdery mildew in rubber trees. By employing six distinct machine learning model construction methods, with the disease index of powdery mildew in rubber trees as the response variable and spore concentration, temperature, humidity, and infection time as predictive variables, a preliminary predictive model for the disease index of rubber-tree powdery mildew was developed. Results from indoor inoculation experiments indicate that spore concentration directly influences disease progression and severity. Higher spore concentrations lead to faster disease development and increased severity. The optimal relative humidity for powdery mildew development in rubber trees is 80% RH. At varying temperatures, the influence of humidity on the disease index differs across spore concentration, exhibiting distinct trends. Each model effectively simulates the progression of powdery mildew in rubber trees, with predicted values closely aligning with observed data. Among the models, the Kernel Ridge Regression (KRR) model demonstrates the highest accuracy, the R^2^ values for the training set and test set were 0.978 and 0.964, respectively, while the RMSE values were 4.037 and 4.926, respectively. This research provides a robust technical foundation for reducing the labor intensity of traditional prediction methods and offers valuable insights for forecasting airborne forest diseases.

## 1. Introduction

Rubber trees (*Hevea brasiliensis*) have significant economic value in southern China and are widely distributed in provinces such as Hainan, Yunnan, and Guangdong [1,2]. The powdery mildew of rubber trees, caused by *Erysiphe quercicola*, is the disease with the largest occurrence area and the highest potential for harm in the main rubber-production areas of our country [3]; it mainly infects the young tissues of rubber trees, and in severe cases, it leads to large-scale leaf drop, directly causing a significant loss in the output of natural rubber and it has emerged as a serious threat to rubber-growing regions [4,5,6]. Powdery mildew of rubber trees is a typical airborne fungal disease, the occurrence and prevalence of which are mainly restricted by three factors: host phenology, climatic conditions, and the initial pathogen concentration; its severity and prevalence rate are closely related to meteorological conditions [7,8]. Temperature and humidity directly affect the germination and growth and development of the conidia of powdery mildew of rubber trees, and ultimately affect the disease index in the field [9]. The germination conditions of the conidia of the powdery mildew are relatively broad; controlled indoor experiments have shown that the germination rate is also relatively high under the conditions of 15–20 °C and low humidity, as well as 28 °C and high humidity—at a temperature of 20–30 °C, the airborne conidia deposited on the young leaves of rubber trees can infect rubber trees within 24 h [10,11]. Powdery mildew can be found in rubber-tree fields throughout the year through the spread of conidia in the air. It is particularly frequent in winter and spring, and the spread rate is even faster when the temperature is low and the humidity is high. It can quickly break out into a disaster in a short time, seriously threatening the stable development of the rubber industry.

Traditional prediction methods for powdery mildew primarily depend on experienced professionals who regularly monitor field-incidence data in rubber plantations to formulate disease-control strategies; however, this approach is labor-intensive, highly subjective, and associated with time-consuming and physically demanding field surveys that may yield imprecise sampling; moreover, traditional predictive analysis can only be conducted after the tender leaves have already been infected, resulting in a lack of timeliness in disease forecasting [12,13]. In recent years, disease prediction models based on computer technology and mathematical statistics have been widely applied, achieving excellent results that surpass traditional prediction and forecasting methods [14]. They can complement and correct traditional forecasting and reporting methods, which is more conducive to reducing the labor intensity of grassroots forecasting and reporting personnel. In recent years, machine learning methods have experienced rapid development. Their integration with plant disease prediction and early warning systems has yielded outstanding results that surpass traditional prediction approaches [15]. The algorithms successfully applied in plant disease prediction and early warning models primarily include artificial neural networks (ANNs), random forests (RFs), and support vector machines (SVMs), and the prediction accuracy of these models varies depending on the specific factors and conditions under which they are applied [16,17,18]. With the growing prevalence of extreme climates globally, such as El Niño–Southern Oscillation (ENSO), and the increasing frequency of extreme events like disease outbreaks and secondary leaf drop in plants, the challenge of predicting plant disease using predictive models continues to rise [19,20,21].

Therefore, in the process of monitoring and employing early warning systems for the occurrence and development of powdery mildew in rubber trees, there is an urgent need to predict the powdery mildew index of rubber trees by integrating climatic variables with the spore concentration of powdery mildew. Based on indoor inoculation experiments, this study aims to (1) quantitatively analyze the effects of spore concentration, temperature, relative humidity, and infection duration on the progression of rubber-tree powdery mildew. (2) Develop prediction models for the rubber-tree powdery mildew disease index by incorporating spore concentration and meteorological factors based on machine learning. The results will deepen the understanding of how spore concentration and meteorological factors influence the severity of powdery mildew in rubber trees, provide theoretical support for the development of prediction and early warning technologies for significant diseases in other economic forest trees, and reduce the challenges and costs associated with researching powdery mildew prediction and early warning systems.

## 2. Results

### 2.1. The Impact of Humidity on the Disease Index

**Figure 1:**
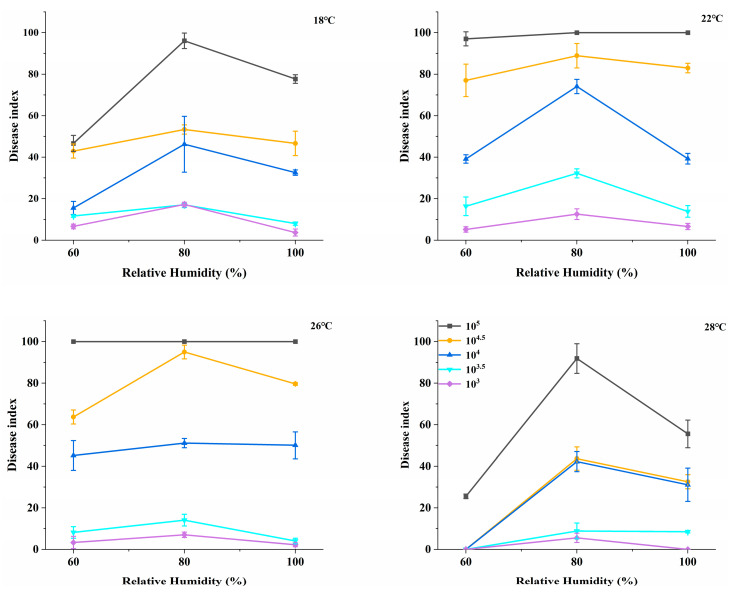
The influence of different humidity levels on the disease index (DI) at 7 days post-inoculation. Data represent mean DI ± SE across biological replicates (*n* = 3, each comprising three rubber seedlings with 10 leaves sampled per seedling). Disease index was calculated as described in Materials and Methods (Formula (2)). Invisible error bars indicate complete consistency among replicates (SE = 0).

Under varying temperature and humidity conditions, powdery mildew was inoculated, and disease development was monitored. At 80% relative humidity (RH), the disease index (DI) was highest across different temperatures and spore concentrations (Figure 1). Therefore, it can be concluded that 80% RH represents the optimal humidity condition for the development of powdery mildew in rubber trees under specific temperatures and inoculation spore concentrations.

### 2.2. The Influence of Spore Concentration, Temperature, Humidity, and Inoculation Timing on the Disease Index of Powdery Mildew in Rubber Trees

**Figure 2:**
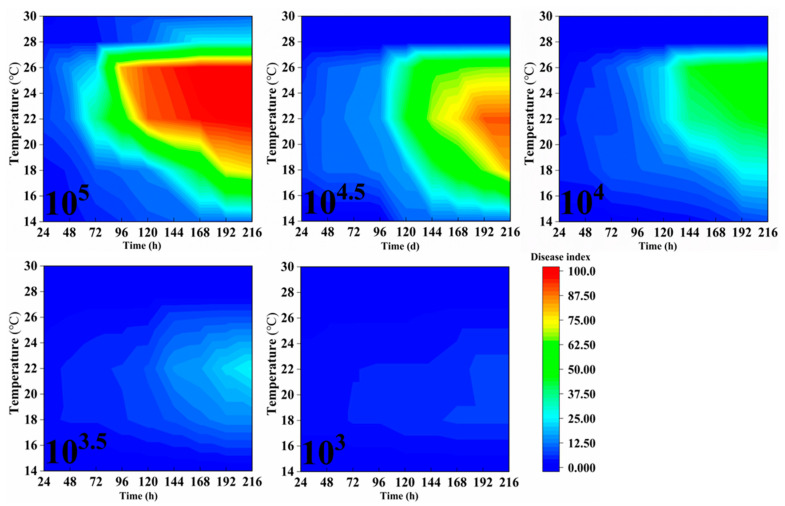
The dynamic impact of spore concentration, temperature, humidity, and inoculation timing on the disease index of powdery mildew in rubber trees at 60% RH. Data represent mean DI across biological replicates, data collection, and DI calculation consistent with Figure 1.

**Figure 3:**
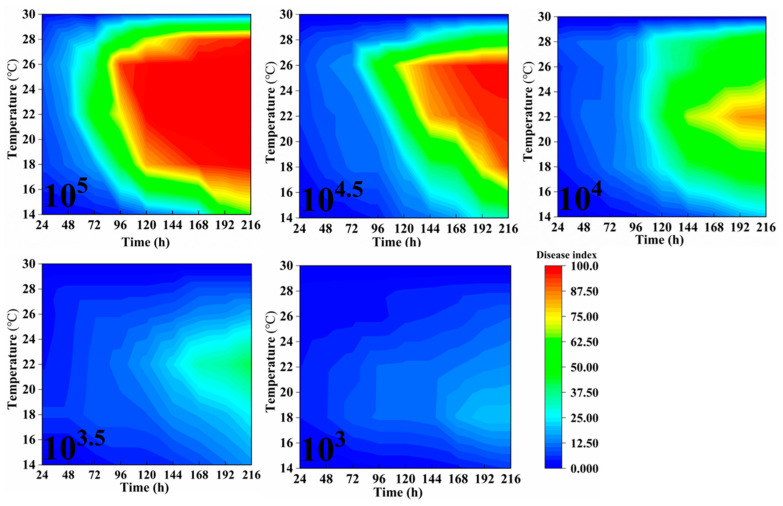
The dynamic impact of spore concentration, temperature, humidity, and inoculation timing on the disease index of powdery mildew in rubber trees at 80% RH. Data collection and DI calculation are consistent with Figure 1.

**Figure 4:**
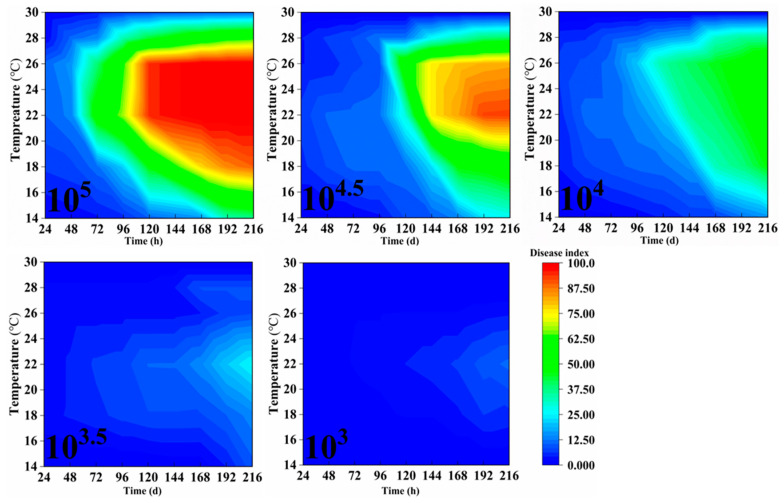
The impact of spore concentration, temperature, humidity, and inoculation timing on the disease index of powdery mildew in rubber trees at 100% RH. Data collection and DI calculation are consistent with Figure 1.

The bronze-stage leaves of rubber trees were inoculated with varying spore concentrations and cultivated under different temperature and humidity conditions. Beginning 24 h post-inoculation, the disease grades of 10 leaves from the middle portion of each tree were observed and recorded daily, and the disease index was calculated accordingly. Observations were continued for 9 consecutive days, with the results presented in Figure 2, Figure 3 and Figure 4. Indoor inoculation experiments have shown that the disease index of powdery mildew on rubber trees is significantly affected by spore concentration, temperature, relative humidity and infection time. The higher the spore concentration, the faster the disease progresses, the shorter the incubation period of the disease, and the higher the final disease index.

Moreover, a significant threshold effect is observed when the spore concentration is ≥10^4^. Under suitable conditions (22–26 °C, RH ≥ 60%), when the spore concentration is ≥10^4^, the disease index can rapidly rise above 60 within 4–7 days after inoculation. Under low-temperature conditions (14–16 °C), the disease development was significantly delayed, and the disease index remained below 50 within 9 days after inoculation. However, at 30 °C, pathogen growth was completely inhibited, and no disease was observed after inoculation, which might be related to the accelerated aging of leaves due to excessively high temperatures or the inactivation of pathogenic bacteria caused by high temperatures. Under high humidity conditions (80–100% RH), powdery mildew outbreaks are promoted. Especially at 22–26 °C, the disease index reaches above 70 within 5 days. The disease progression is slightly slower at 60% RH, but when the spore concentration is ≥10^4.5^, the disease index can still exceed 75 in 9 days post-inoculation (Figure 2, Figure 3 and Figure 4).

**Figure 5:**
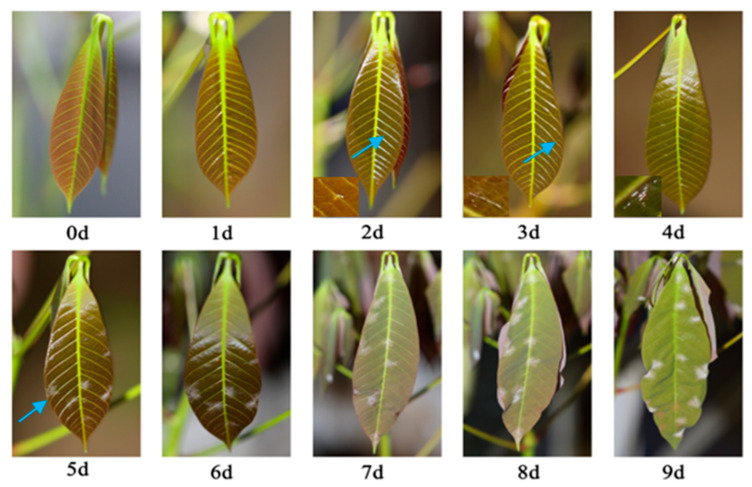
The dynamic variation in disease with infection time at 22 °C and 80% RH under a spore concentration of 10^4^. The blue arrows mark the early lesions that are difficult to identify with the naked eye.

Complementing the disease index trends presented in Figure 2, Figure 3 and Figure 4, Figure 5 demonstrates a sequential visual record of symptom progression under representative experimental conditions; these images clearly illustrate the pattern and extent of symptom development over time. In the early stages after inoculation, transparent mycelium formation can be observed at the inoculation site of the leaves, corresponding to initial disease grade values of grade 0–1 (within 0–4 d); subsequently, the mycelium expands in large numbers and forms a lesion with each other, coinciding with the steep disease grade increase to grade 5–7 (within 5–7 d); the disease grade and disease index gradually worsen, and even phenomena such as leaf shrinkage occur, rapidly promoting the upgrading of the powdery mildew disease grade (within 8–9 d) to peak grade 9 (Figure 5).

**Figure 6:**
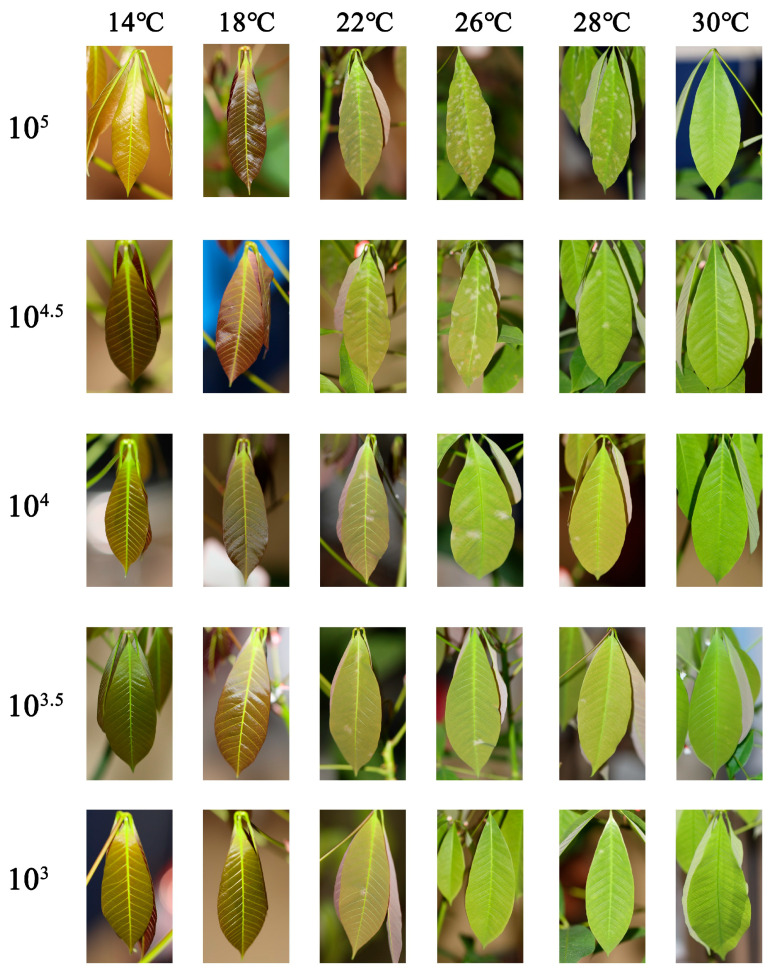
The impact of varying spore concentrations and temperatures on the disease grade assessed at 7 days post-inoculation at 80% RH.

Figure 6 illustrates that assessing the disease grade 7 days after inoculation, it was observed that the development of powdery mildew was influenced by both temperature and spore concentration, exhibiting a temperature-dependent pattern and a concentration threshold effect. Within the temperature range of 22–26 °C, all spore concentration treatments resulted in the highest disease grades. When the temperature exceeded 26 °C or dropped below 22 °C, the disease grade decreased significantly: At the 80% RH, under high spore concentration conditions (10^5^ spores/mL), the disease grade reached grade 9 within the temperature range of 22–28 °C. At 18 °C, the disease grade was 7, while at 14 °C and 30 °C, it was only grade 1 and grade 3, respectively. At a spore concentration of 10^4.5^ spores/mL, the disease grade reached grade 9 at 26 °C, grade 7 at 22 °C, and grade 5 at 28 °C, with only grade 1 observed under other temperature conditions. At a spore concentration of 10^4^ spores/mL, the disease grade reached grade 5 at both 22 °C and 26 °C, while the disease grade at other temperatures remained at grade 3. Under relative lower spore concentration conditions (10^3^ and 10^3.5^ spores/mL), the disease grades across all temperature treatments remained relatively low (grades 1–3). Visual-graphic consistency validated our quantitative measurements of the dynamic environmental effects on disease index progression.

### 2.3. The Correlations Between Spore Concentration, Temperature and Humidity, Infection Time and Disease Index

Table 1 presents the results of a single-factor effect test for four variables (spore concentration, humidity, temperature, and infection time) on the disease index. The results show that all four factors significantly influence the disease index, with *p*-values less than 0.001. Spore concentration had the largest impact with an f-value of 194.51 followed by temperature (F = 112.53), humidity (F = 27.39) and infection time (F = 66.77). These significant F-values indicate that changes in these variables correspond to substantial variations in the disease index. For example, the disease index increases with spore concentration, with higher concentrations correlating with higher disease index values. Similarly, temperature and humidity also show clear patterns where higher values of these factors lead to increases in the disease index, although the temperature exhibits a more complex relationship. The significant impact of infection time on the disease index suggests that the longer the exposure, the higher the disease index, which supports the importance of time in the disease progression.

Based on linear regression analysis (Table 2), we further investigate how these variables affect the disease index in a more refined manner. The results show that all predictors are statistically significant. Spore concentration has the strongest positive impact on the disease index, Temperature has a negative relationship with the disease index (B = −0.181, *p* = 0.018), which suggests that higher temperatures may reduce the disease index. Humidity and infection time also significantly contribute to the disease index (B = 0.098, *p* < 0.001, and B = 0.182, *p* < 0.001, respectively). The model explains 40.2% of the variation in the disease index (R^2^ = 0.402) and the highly significant F-value (F = 425.259, *p* < 0.001) indicates that the regression model as a whole is very effective. Additionally, the variance inflation factors (VIFs) and tolerance values suggest that multicollinearity is not a concern. Notably, interaction effects were observed among these variables, with both binary and ternary combinations showing highly significant impacts on the disease index (*p* < 0.001) (Table 3). This indicates that the four variables collectively influence the final disease index.

### 2.4. Model Hyperparameter Tuning

This study employs a grid search to systematically optimize the key hyperparameters of each predictive model, aiming to achieve an optimal balance between model performance and computational efficiency. The goal is to establish a reliable model configuration for constructing the rubber tree powdery mildew disease index prediction model.

**Figure 7:**
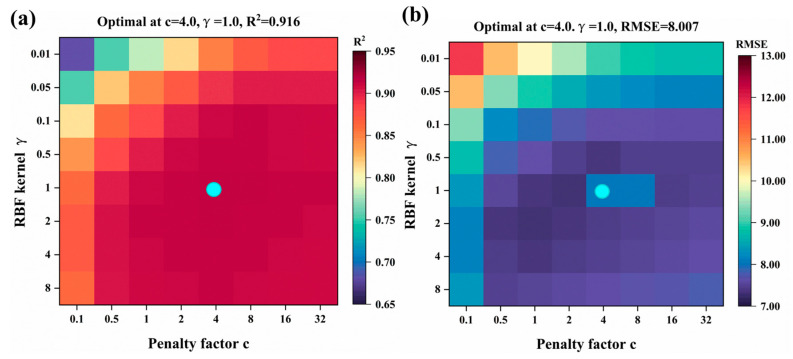
Optimizing SVM model hyperparameters by grid search in test set: (**a**) R^2^; (**b**) RMSE. The blue dots represent the optimal parameters of the model.

In the support vector machine (SVM) model, the radial basis function (RBF) is selected as the kernel function, with its mathematical expression provided in Equation (1). Following optimization, the penalty factor C was set to 4.0 and the kernel hyperparameter *γ* to 1.0 (Figure 7). This hyperparameter combination effectively balances model complexity and generalization capability. The RBF kernel function, which computes similarity based on sample distances, ensures high computational efficiency when applied to large-scale agricultural datasets.

(1) $$K\left(x_{i},x_{j}\right)=\exp\left(-\gamma ∥x_{i}-x_{j}{∥}^{2}\right)$$

$x_{i}$ and $x_{j}$ are sample vectors, and *γ* is a hyperparameter of the kernel function.

**Figure 8:**
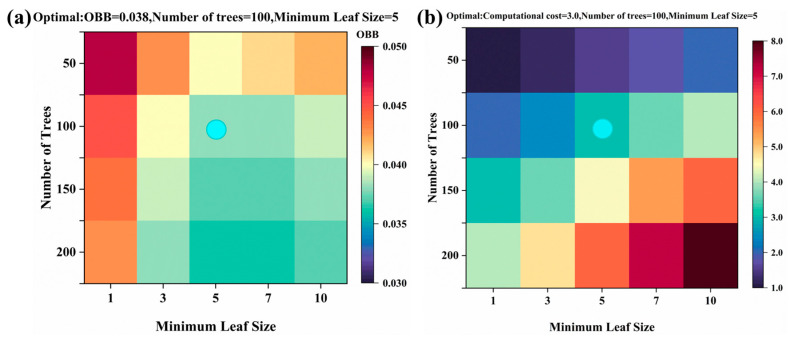
Optimizing RF model hyperparameters by grid search in test set: (**a**) OBB; (**b**) computational cost. The blue dots represent the optimal parameters of the model.

For the random forest (RF) model, optimization is carried out by constructing a two-dimensional hyperparameter space that includes the number of decision trees and the minimum number of leaves, where the search range for the number of decision trees is [50, 100, 150, 200], and the search range for the minimum number of leaves is [1, 3, 5, 7, 10]. Taking the out-of-bag error (OOB error) as the evaluation index and with computational cost being monitored concurrently, number of decision trees = 100 and minimum leave size = 5 was finally selected as the optimal hyperparameter combination. This combination achieves the lowest OOB error (0.038) and moderate computational cost (3.0) on the test set, preventing overfitting while maintaining model sensitivity (Figure 8). The hyperparameter optimization results show that when the number of decision trees is below 100, the model tends to underfit; conversely, increasing the number beyond 100 yields limited performance gains while significantly raising computational costs. Additionally, setting the minimum number of leaves below 5 may lead to overfitting, whereas a value above 5 can reduce the model’s sensitivity. This hyperparameter selection effectively regulates the complexity of decision tree growth, thereby mitigating the risk of overfitting to the training data while preserving the model’s robust generalization capability for new data.

**Figure 9:**
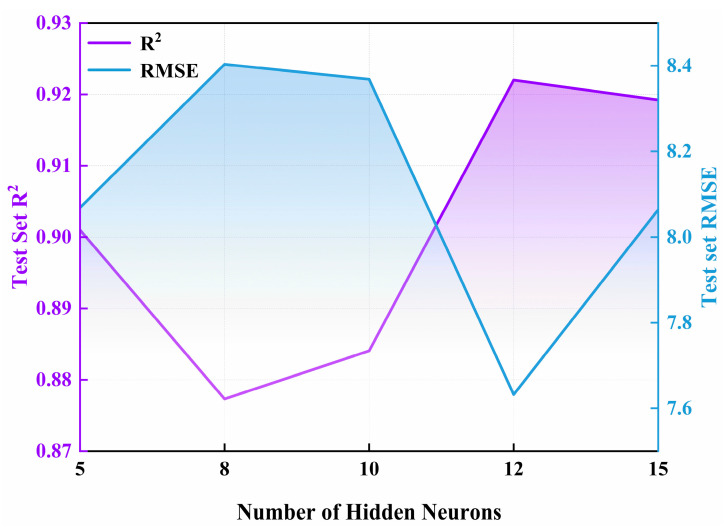
Optimizing ANN model hyperparameters in test set.

The artificial neural network (ANN) model constructed in this study adopts a three-layer structure. The number of neurons in the hidden layer is determined to be 12. The model training uses a hyperparameter setting of up to 500 iterations, with the error threshold set at 10^−6^ and the learning rate set at 0.005. These configurations ensure the complete convergence and stability of model training. At this point, the R^2^ of the test set reaches 0.922, and both the RMSE values of the test set remain stable and relatively low (Figure 9).

**Figure 10:**
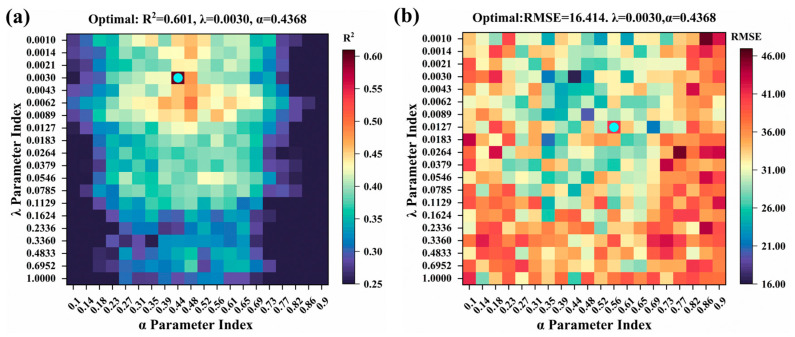
Optimizing Elastic Net model hyperparameters by grid search in test set: (**a**) R^2^; (**b**) RMSE. The blue dots represent the optimal parameters of the model.

**Figure 11:**
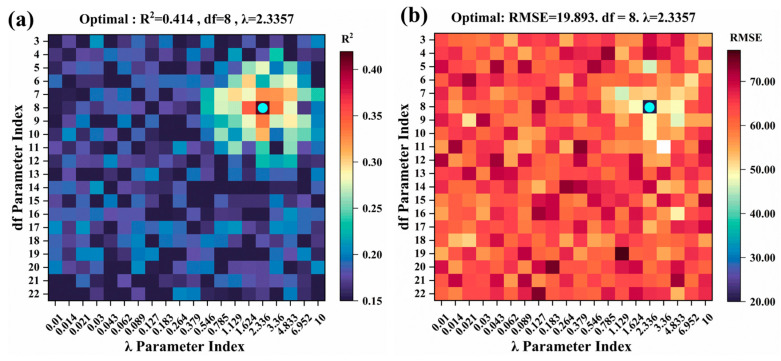
Optimizing GAM hyperparameters by grid search in test set: (**a**) R^2^; (**b**) RMSE. The blue dots represent the optimal parameters of the model.

The hyperparameter configuration for the Elastic Net model is set at α = 0.4368 and λ = 0.003 (Figure 10), the R^2^ value of the test set is relatively low at 0.601. In contrast, the Generalized Additive Model (GAM) was configured with λ = 2.3357, df = 8 (Figure 11), resulting in a test set R^2^ of 0.414, which represents the weakest performance among the models evaluated.

**Figure 12:**
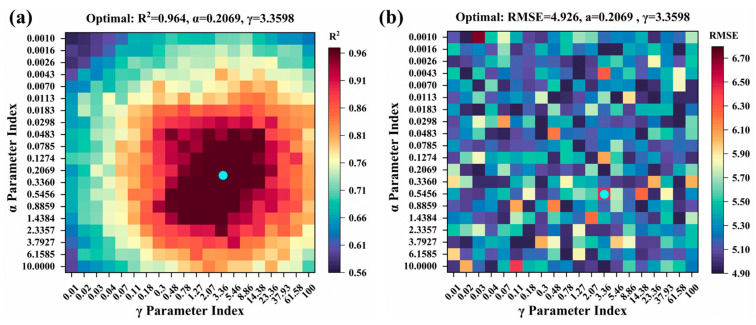
Optimizing KRR model hyperparameters by grid search in test set: (**a**) R^2^; (**b**) RMSE. The blue dots represent the optimal parameters of the model.

The KRR model based on the RBF kernel function selects the hyperparameter combination of *γ* = 3.3598 and α = 0.2069, which exhibits the highest predictive performance (test set R^2^ = 0.964) and effectively captures the nonlinear interaction patterns present in the data (Figure 12).

The hyperparameter selection of each model has been systematically verified, ensuring the scientific and rationality of the model configuration. Among them, the KRR model based on the RBF kernel function has a more outstanding prediction performance, while the prediction effects of the Elastic Net model and the GAM are relatively weak.

### 2.5. Comparative Analysis of Different Prediction Models for Rubber-Tree Powdery Mildew Disease Index

**Figure 13:**
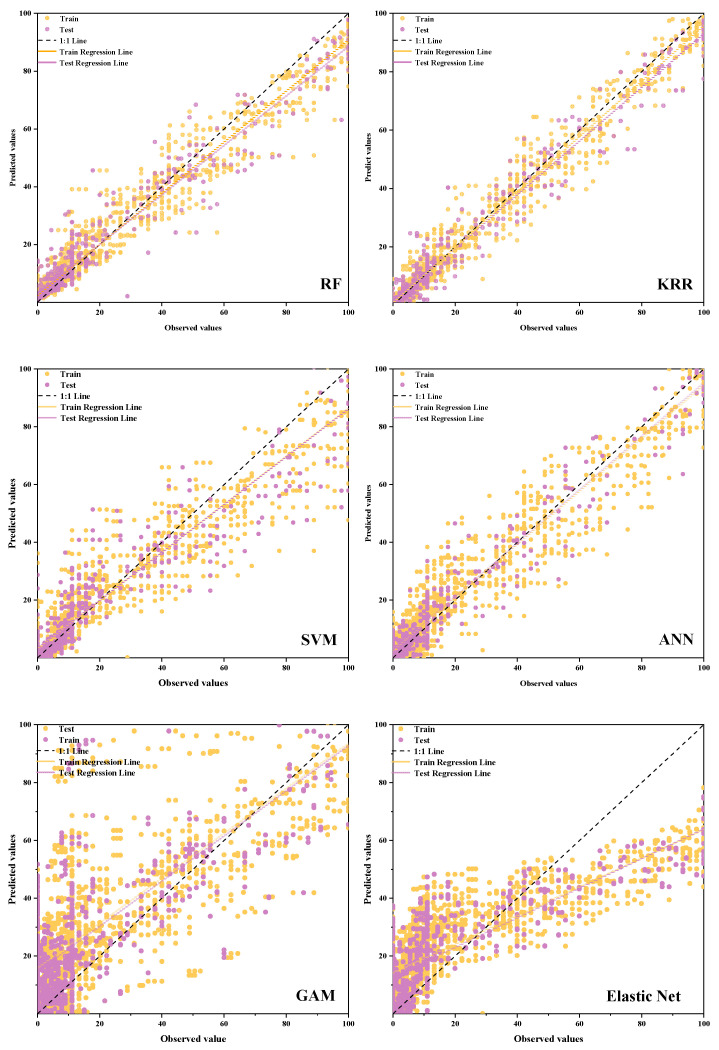
A comparison of the observed values and predicted values of different prediction models.

Figure 13 and Table 4 illustrates the performance of models derived from different modeling methods. The prediction accuracy of the training and test sets varies across these methods. Notably, the KRR model demonstrated the best predictive performance, with R^2^, RMSE, MAE, and MBE values for the training set being 0.978, 4.037, 2.389, and 0.272, respectively, and those for the test set being 0.964, 4.926, 2.880, and 0.336, respectively. These results indicate no significant difference between the training and test sets. Furthermore, the fitted regression line between the actual and predicted values of the disease index is closest to the 1:1 trend line, with the predicted values distributed evenly on both sides of the regression line. This suggests that the overall trend is highly reliable. Therefore, the KRR model exhibits strong stability and generalization capability in predicting the disease index of rubber-tree powdery mildew, offering considerable accuracy for subsequent prediction model applications.

The mean bias error (MBE) quantifies systematic bias in predictive models. An MBE approaching zero indicates high accuracy, whereas positive/negative values reveal consistent overestimation or underestimation. In Elastic Net and GAM, the MBE values for the training set were −4.240 and −11.440, respectively, while those for the test set were −4.381 and −10.849, respectively. These results indicate that both models systematically underestimated the true value when predicting the powdery mildew disease index. Additionally, the R^2^ values for the training set were 0.612 and 0.412 for Elastic Net and GAM. For the test sets, the R^2^ values were 0.601 and 0.414, respectively, and the RMSE values were 16.414 and 19.893, respectively. These metrics confirm that the two models performed relatively poorly, with predicted values being discretely distributed around the 1:1 trend line, indicating significant underestimation.

Overall, the KRR, RF, ANN, and SVM models demonstrated strong predictive capabilities, with their prediction results aligning to some extent with the incidence characteristics of powdery mildew under low-temperature and high-humidity conditions, and the Elastic Net model and the GAM exhibited relatively weak predictive abilities in this experiment. Based on the prediction hyperparameters of the rubber-tree powdery mildew disease index, the fitting accuracies of the six models can be ranked as follows: KRR > RF > ANN > SVM > Elastic Net > GAM.

## 3. Discussion

The indoor inoculation experiments demonstrated a highly significant correlation (*p* < 0.01) between the spore concentration, temperature, relative humidity, and infection time of rubber-tree powdery mildew and the disease index of rubber-tree powdery mildew. There was an interaction effect among various environmental factors, infection time, and spore concentration on the disease index. As the concentration of inoculated spores increased and the infection time extended, the disease index correspondingly increased. A spore count of 10^5^ could result in a disease index of 100 at temperatures ranging from 18 to 28 °C. When the spore concentration was 10^4^, the disease index reached 100 only at 22 °C. When the spore concentration was ≤10^3.5^, under different combinations of temperature and humidity conditions, the maximum disease index did not exceed 30, and the number of lesions was very small. These results indicate that the spore concentrations of rubber-tree powdery mildew directly affect the progression rate of the disease course and the final disease index, which aligns with field observation reports.

An appropriate temperature enhances the germination rate of conidia of rubber-tree powdery mildew and the expansion points of disease spots on the leaf surface, thereby accelerating the expansion speed of disease spots. Temperatures exceeding 30 °C or dropping below 14 °C are not conducive to the exponential growth of powdery mildew in rubber trees. When the temperature is between 22 and 26 °C, the disease index of rubber-tree powdery mildew increases rapidly and reaches a relatively high level 216 h after inoculation. It is found that when the temperature is ≥30 °C or ≤15 °C, the disease index remains relatively low; it was concluded that the optimal germination temperature is 23.2 °C, which is consistent with the findings of this study [11]. At high inoculation concentrations, there is no significant difference in the disease course at temperatures ranging from 22 to 26 °C. However, as the inoculation concentration decreases, the disease course progresses more rapidly at 22 °C, potentially due to the spore germination rate. Ultimately, spore supersaturation leads to a high disease index. Relative humidity also influences disease progression; high relative humidity has a relatively smaller effect on disease progression. The disease index progresses fastest when the relative humidity is 80%. When the temperature is high (28 °C or above) and the relative humidity is low (60% RH), the development of the powdery mildew disease index in rubber trees will be delayed. A study demonstrated that the maximum severity of tomato powdery mildew occurred at 80% relative humidity (RH) and gradually decreased as RH increased (up to 95% RH), which aligns with the findings of this study [22]. In onion-growing regions of Quebec, Australia, spore traps were used to collect spores of various onion diseases, and predictive models were employed to provide control thresholds, offering advice and references for precise control, and reducing pesticide application frequency and the risk coefficient by 28% [23]. Another study demonstrated that meteorological conditions are strongly correlated with the concentration of fungal spores [24]; consequently, integrating meteorological conditions with spatiotemporal variations in spore concentrations can enhance our understanding of the variation patterns of the powdery mildew disease index in rubber trees, thereby facilitating the development of a more accurate disease index prediction model.

Traditional statistical methods, such as stepwise linear regression, multivariate linear regression, and logistic regression, have exhibited certain limitations in disease prediction [25]. Machine learning models have demonstrated the ability to simulate complex nonlinear relationships and explore the potential relationships among various variables more effectively than other algorithms, exhibit superior prediction accuracy compared to traditional statistical methods [26,27].

In this study, the prediction performance of the Elastic Net model and the GAM was significantly lower than that of the nonlinear methods (KRR, ANN, RF, SVM). The analysis of the existing results indicates that the predictive performance of the GAM and the Elastic Net is relatively insufficient, which may be related to the potential mismatch between the structural characteristics of their models and the nonlinear requirements of the data. Although GAM processes univariate nonlinear relationships through smoothing functions, its additive structure cannot effectively characterize the complex interactions among multiple variables (such as the coupling effect of spore concentration, temperature, and relative humidity), resulting in the insufficient capture of the high-order dynamic characteristics of powdery mildew index [28]. The linear nature of Elastic Net makes it even more difficult for them to adapt to real nonlinear relationships; even if polynomial features are introduced, they still cannot fully simulate biological processes such as threshold effects [29]. This structural defect is manifested in indicators as a significant systematic underestimation (MBE: −4.240 to −11.440) and low explanatory power (R^2^ ≤ 0.601). Furthermore, the limitations of model performance are closely related to the way data features are processed, both types of models are limited by the insufficient number of predictor variables, which may omit key environmental factors (such as light intensity) or host physiological indicators, further weakening the completeness of the models [30]. The epidemiology of powdery mildew involves complex spatiotemporal interaction effects (such as the dynamic coupling of temperature, humidity, and time), while the existing models only incorporate some explicit interaction terms (such as spore concentration × temperature, etc.), and fail to fully analyze the multi-dimensional nonlinear synergy. The insufficiency of this interaction modeling directly leads to the discrete distribution of predicted values (Elastic Net model test set RMSE = 16.414). Meanwhile, the limitations of data collection may overlook potentially important covariates, such as crop canopy microclimate or pathogen genetic diversity indicators, resulting in structural deficiencies in model input information [31,32]. Two improvement paths need to be adopted for future research: First, develop hybrid architectures (such as GAM with tensor product smooths) to explicitly model multi-scale interactions; secondly, by integrating high-throughput phenomics and other technologies, the dimensions of predictor variables should be expanded to ensure that the data features match the complexity of the model [33,34]. These measures will significantly enhance the ability to analyze the complex systems of plant diseases.

In contrast, the top-performing KRR model (test set R^2^ = 0.964) confirms the advantages of kernel methods in modeling nonlinear relationships, as it breaks through the linear or additive constraints of traditional models through implicit high-dimensional mapping, avoiding the limitations of manually designing interaction terms [35,36]. Other prominent nonlinear methods also exhibit distinct advantages: the ANN model automatically learns high-order feature interactions through its multi-layer perceptron architecture with nonlinear activation functions [37]; the RF model employs an ensemble of decision trees to support nonlinear relationships and feature interactions via recursive partitioning [38]; and the SVM model maps data into high-dimensional spaces using kernel techniques to separate complex decision boundaries [39]. The shared strength of these methods lies in their data-driven modeling approach, which enables them to adapt automatically to complex dependencies in the data without requiring predefined specific relationship forms between features. This insight provides a valuable reference for model selection in similar tasks. *Sclerotinia sclerotiorum* incidence rate models were constructed based on factors such as temperature, leaf wetting duration, and inoculation time through ANN, decision trees (DTs), and SVM, with the accuracy of the ANN exceeding 89% [40]. An ANN model was developed using temperature and humidity duration as input features to predict powdery mildew infection in ryegrass, and it was confirmed that the probability of infection significantly decreased when the temperature surpassed a specific threshold [41]. Short-term and long-term prediction models for the disease index of rubber-tree powdery mildew were developed using RF and ANN, and the highest temperature was identified as the most important feature among the short-term models [42].

This study developed a dynamic prediction model for rubber-tree powdery mildew using various approaches. However, this study is based on data obtained under controlled laboratory conditions, which may constrain the potential performance and practical applicability of the prediction model in dynamic field environments. These limitations could arise from interference by various environmental factors, such as solar radiation, ultraviolet radiation, rainfall, wind speed, and host-leaf phenology.

The prediction model developed in this study provides a foundation for the development of an intelligent monitoring system for powdery mildew on rubber trees. Future research will involve data collection through the deployment of field Internet of Things (IoT) devices: real-time acquisition of spore concentration dynamics via spore capture terminals; real-time monitoring of canopy micro-environmental conditions (temperature, relative humidity, leaf-surface wetness duration) using field sensors; and retrieval of host phenological stages through multispectral NDVI data obtained from unmanned aerial vehicles (UAVs). These indicators will be incorporated as predictive variables into this prediction model, thereby enhancing the accuracy and applicability of the model [43,44]. Meanwhile, the identification and detection of plant leaf lesions is an effective method for predicting and forecasting plant diseases, and it has different established patterns in different crops, including different crops such as apples, corn, grapes, peppers, potatoes, and tomatoes [45,46]. Our subsequent research will focus on the automatic identification of powdery mildew on rubber tree leaves and the classification of disease grade, aiming to cross-validate the prediction results of our existing disease index model. Additionally, GIS technology, fluid mechanics, and aerobiology could be integrated to investigate the spatiotemporal dynamic distribution patterns of regional spore release, diffusion, and propagation at various scales [47,48,49]. These approaches would provide effective support for guiding green prevention and control strategies as well as unified prevention and control measures for rubber-tree powdery mildew, holding significant practical implications for managing airborne fungal diseases affecting forest crops.

## 4. Materials and Methods

### 4.1. Materials

The rubber tree ‘Reyan 73397’ bud seedlings used in the experiment were procured from the nursery of the Fifth Germplasm Resources Team at the Rubber Research Institute, Academy of Tropical Agricultural Sciences. Healthy bud seedlings with uniform height and newly emerged leaves were selected and planted in 3-gallon flowerpots filled with peat soil. They were watered with a nutrient solution every two weeks.

The rubber-tree powdery mildew fungus used in this study was collected from the nursery of the Fifth Germplasm Resources Team at the Rubber Research Institute, Academy of Tropical Agricultural Sciences. The strain was isolated through single-spot isolation in our laboratory and subsequently stably cultured. It was identified as HO-73.

To collect spores of the rubber-tree powdery mildew fungus from the leaf surface, a suspension was prepared by mixing 20% (*v*/*v*) of 0.2% Tween 80 with 80% (*v*/*v*) ddH_2_O. The mixture was centrifuged and concentrated at 5000 r/min for 1 min, after which the supernatant was discarded. The spores were resuspended in ddH_2_O and adjusted to a concentration of 10^6^ cells/mL using a hemocytometer. A series of gradient dilutions were then performed to prepare suspensions of powdery mildew spores at various concentrations: 10^5^ spores/mL, 10^4.5^ spores/mL, 10^4^ spores/mL, 10^3.5^ spores/mL, and 10^3^ spores/mL.

### 4.2. Experiment on the Effects of Environmental Factors and Spore Concentration on the Disease Progression of Powdery Mildew in Rubber Trees

The powdery mildew spores with five concentration gradients of 10^5^ spores/mL, 10^4.5^ spores/mL, 10^4^ spores/mL, 10^3.5^ spores/mL, and 10^3^ spores/mL were inoculated into the healthy young leaves of ‘Reyan 73397’ by the spray method and cultivated in an artificial climate chamber with constant temperature and humidity. Three relative humidity levels (60% RH, 80% RH, 100% RH) and six temperature gradients (14 °C, 18 °C, 22 °C, 26 °C, 28 °C, 30 °C) were set for combination to obtain 90 experimental treatments, and each experimental treatments were conducted with three replicates, and three rubber bud seedlings were inoculated in each replicate, resulting in a total of 810 seedlings across all treatments. For each seedling, disease assessment was performed on 10 marked middle leaves. The light intensity was set to 10,000 LX, with light exposure: darkness = 14 h:10 h. The disease grades of each trial treatment were independently evaluated at each time point (24 h, 48 h, 72 h, 96 h, 120 h, 144 h, 168 h, 192 h, and 216 h post-inoculation), and the disease index (DI) was calculated.

Each seedling’s disease evaluation was conducted independently, the three biological replicates under identical experimental conditions were strictly separated in both space and time to prevent any potential interference or contamination.

### 4.3. Grading Standards and Calculation Methods of Disease Index for Rubber-Tree Powdery Mildew Leaves

The reference criteria for leaf grading of rubber-tree powdery mildew and the calculation method of the disease index are specified in “NY/T 1089-2015 Technical procedure for forecasting the powdery mildew of rubber tree” (Ministry of Agriculture and Rural affairs of the People’s Republic of China, 2015), as detailed in Table 5 and Formula (2) [50].

The calculation formula for the disease index of rubber-tree powdery mildew:

(2) $$DI=\frac{\sum Nd\times Dg}{N\times Dm}$$

In Equation (2), DI is the disease index, Nd is the number of diseased leaves at each disease grade, Dg denotes the corresponding disease grade, N is the total number of leaves investigated, and Dm is the representative value at the highest level.

### 4.4. Prediction Model Construction and Model Hyperparameter Tuning

This study utilizes the following six machine learning algorithms to develop predictive models: KRR [51], RF [52], ANN [53], SVM [54], Elastic Net [55], and GAM [56], taking the spore inoculation concentration, temperature, humidity, and inoculation time as predictive variables and the disease index as response variables. All experimental data collected and detailed in Section 4.2 were utilized for analysis and randomly allocated in an 8:2 ratio, that is, 80% of the data was used as the training set for model fitting, and 20% of the data was used as the test set for model checking to ensure the independence among the samples.

Prior to model training, all input features are normalized through linear transformation to the [0, 1] range (Formula (2)). This step mitigates the impact of dimensional discrepancies on the training process. During the model prediction phase, the output results undergo inverse normalization to recover their original physical meaning (Formula (3)). All performance metrics are computed based on the inversely normalized data to ensure that the evaluation results carry clear and meaningful physical interpretations.

(3) $$X_{norm}=\frac{X-X_{min}}{X_{max}-X_{min}}$$

(4) $$X_{denorm}=X_{norm}\times \left(X_{max}-X_{min}\right)+X_{min}$$

*X* denotes each original disease index value; *X_min_* and *X_max_* denote the minimum and maximum values of the original data, respectively; *X_norm_* represents the normalized input value of the model, while *X_denorm_* refers to the actual output value.

The grid search method was systematically applied to optimize the key hyperparameter of each predictive model, with the objective of achieving a balance between model performance and computational efficiency. This approach ensured the selection of reliable model configurations for the development of the rubber-tree powdery mildew disease index prediction model. The final hyperparameter configurations for each model are summarized as follows:

(1) The KRR model maps input features into a high-dimensional feature space using the RBF kernel function with *γ* = 3.3598, while incorporating a regularization hyperparameter α = 0.2069 to balance model fitting and generalization performance.
(2) The SVM model selected the RBF kernel function with hyperparameters (C = 4.0, *γ* = 1.0).
(3) For the RF model, the number of decision trees was set to 100, and the minimum leaf size was set to 5.
(4) The ANN model adopted a single-hidden-layer structure containing 12 neurons, with this configuration determined by balancing performance between the training and test sets.
(5) The Elastic Net model determined the optimal hyperparameters by setting α = 0.4368 (representing the L1/L2 regularization weight ratio) and λ = 0.003.
(6) The GAM captured nonlinear trends in individual features using spline functions (with a smoothing hyperparameter λ = 2.3357, df = 8) and integrated these into the interaction terms.

### 4.5. Prediction Model Performance Evaluation

Run the model with the selected hyperparameters, compare the fitting accuracy of each model, and determine the optimal model hyperparameters. Four types of indicators were adopted to evaluate the model effect: the coefficient of determination R^2^ between the predicted values and the observed values (Formula (5)), the root mean square error RMSE (Formula (6)), the mean absolute error MAE (Formula (7)), and the mean bias error (Formula (8)) to assess the accuracy of the rubber-tree powdery mildew disease index prediction model. The closer R^2^ is to 1 and the smaller the values of RMSE, MAE and MBE are, the better the model’s ability to predict the disease index.

(5) $$R^{2}=1-\frac{\sum_{i}{\left({\hat{y}}_{i}-y_{i}\right)}^{2}}{\sum_{i}{\left(\bar{y_{i}}-y_{i}\right)}^{2}}$$

(6) $$RMSE=\sqrt{\frac{1}{n}\sum_{i=1}^{n}{\left(y_{i}-{\hat{y}}_{i}\right)}^{2}}$$

(7) $$MAE=\frac{1}{n}\sum_{i=1}^{n}\left|\left(y_{i}-{\hat{y}}_{i}\right)\right|$$

(8) $$MBE=\frac{1}{n}\sum_{i=1}^{n}\left(y_{i}-{\hat{y}}_{i}\right)$$

*n* represents the sample size, and $\bar{y}$ represents the mean of the observed values, while $y_{i}$ and ${\hat{y}}_{i}$ correspond to the observed value and the predicted value, respectively.

### 4.6. Statistical Analysis

This study performed statistical analysis using SPSS 27 (IBM, Armonk, NY, USA). Intersubjective effect test was conducted to assess the interaction effects among the various variables. The prediction model was established using Python 3.13.2 (Python Software Foundation, Wilmington, DE, USA). All graphs were generated using Origin 2021 software (OriginLab Corporation, Northampton, MA, USA), with appropriate adjustments for clarity and visual presentation.

## 5. Conclusions

Studies have demonstrated that spore concentration directly influences the progression and severity of powdery mildew in rubber trees. Higher spore inoculation concentrations lead to faster increases in disease severity and greater overall severity. The optimal relative humidity for powdery mildew development in rubber trees is 80% RH. At varying temperatures, the impact of humidity on the disease index differs depending on spore concentration, exhibiting distinct trends. In this study, a machine-learning-based prediction model for rubber-tree powdery mildew was developed, incorporating inoculation spore concentration, temperature, humidity, and inoculation time as key input variables. Among the models evaluated, the KRR model exhibited the highest fitting performance, with R^2^ > 0.96.

## Figures and Tables

**Table 1:** Intersubjective effect test: single-factor effect.

Variable	Diseased Index, Mean ± SD	Statistic	*p*
Total	18.77 ± 27.31		
Spore concentration (lg (Spores/mL))		F = 194.51	<0.001
3	3.36 ± 4.36		
3.5	6.57 ± 7.97		
4	18.96 ± 20.60		
4.5	25.72 ± 30.67		
5	40.26 ± 38.18		
Humidity (%)		F = 27.39	<0.001
60	14.21 ± 24.02		
80	23.76 ± 30.12		
100	18.26 ± 26.53		
Temperature (°C)		F = 112.53	<0.001
14	6.51 ± 10.31		
18	22.20 ± 24.85		
22	32.34 ± 33.69		
26	31.42 ± 34.31		
28	14.98 ± 21.77		
30	1.41 ± 3.21		
Time (h)		F = 66.77	<0.001
24	3.23 ± 3.02		
48	5.04 ± 4.81		
72	7.79 ± 10.19		
96	12.66 ± 18.93		
120	19.44 ± 26.40		
144	25.12 ± 30.78		
168	28.82 ± 32.39		
192	32.65 ± 34.03		
216	34.66 ± 35.08		

**Table 2:** Linear regression.

	Non-standardized Coefficients		Standardized Coefficients	*t*	*p*	Collinearity Diagnosis	
	*B*	Standard Error	*Beta*			VIF	Tolerance
Constant	−80.757	3.687	-	−21.904	0.000 ***	-	-
Spore concentration	18.566	0.601	0.475	30.909	0.000 ***	1.000	1.000
Temperature	−0.181	0.077	−0.036	−2.363	0.018 *	1.001	0.999
Humidity	0.098	0.026	0.058	3.759	0.000 ***	1.001	0.999
Infection time	0.182	0.007	0.413	26.869	0.000 ***	1.000	1.000
*R* ^2^	0.402						
Adjust *R*^2^	0.401						
*F*	425.259 ***						

Note: Dependent variable = disease index. * *p* < 0.05, *** *p* < 0.001.

**Table 3:** Intersubjective effect test: analysis of two-factor interaction and multi-factor interactions.

Two-Factor Interaction	Significance (Two-Tailed)	Multiple Factors Interact	Significance (Two-Tailed)
Spore concentration (log) × Temperature	<0.001	Spore concentration (log) × Temperature × RH	<0.001
Spore concentration (log) × RH	<0.001	Spore concentration (log) × Temperature × Infection time	<0.001
Spore concentration (log) × Infection time	<0.001	Spore concentration (log) × RH × Infection time	<0.001
Temperature × humidity	<0.001	Temperature × RH × Infection time	<0.001
RH × Infection time	<0.001	Spore concentration (log) × Temperature × RH × Infection time	<0.001
Temperature × Infection time	<0.001		

**Table 4:** The performance of different models in the prediction of the disease index.

Model	Train				Test			
	R^2^	RMSE	MAE	MBE	R^2^	RMSE	MAE	MBE
KRR	0.978	4.037	2.389	0.272	0.964	4.926	2.880	0.336
RF	0.957	5.508	3.345	0.048	0.963	5.744	3.659	0.013
ANN	0.919	7.736	5.334	0.899	0.922	7.652	5.440	0.883
SVM	0.897	8.702	4.941	0.051	0.916	8.007	4.757	0.027
Elastic Net	0.612	17.113	13.364	−4.240	0.601	16.414	12.417	−4.381
GAM	0.412	21.075	15.612	−11.440	0.414	19.893	14.301	−10.849

**Table 5:** Classification standards for rubber-tree powdery mildew disease spots.

Disease Grade	Percentage of Spot Area to Leaf Area (x)
Level 0	no powdery mildew spots on the leaf
Level 1	0 < x < 1/20
Level 3	1/20 ≤ x< 1/16
Level 5	1/16 ≤ x < 1/8
Level 7	1/8 ≤ x <1/4
Level 9	x ≥ 1/4

## Data Availability

All data generated or analyzed are included in the article.
